# Predicting outcomes of Lung Cancer using the modified glasgow prognostic score: A systematic review and meta-analysis

**DOI:** 10.12669/pjms.40.3.8397

**Published:** 2024

**Authors:** Yonghua Min, Xiaofeng Li, Huafei Chen, Yumei Xu, Gang Lan

**Affiliations:** 1 Yonghua Min, Department of Chest Disease Center, Zhejiang Rongjun Hospital, 309 Shuangyuan Road, Jiaxing, Zhejiang Province 314000, P.R. China; 2 Xiaofeng Li, Department of Chest Disease Center, Zhejiang Rongjun Hospital, 309 Shuangyuan Road, Jiaxing, Zhejiang Province 314000, P.R. China; 3 Huafei Chen, Department of Chest Disease Center, Zhejiang Rongjun Hospital, 309 Shuangyuan Road, Jiaxing, Zhejiang Province 314000, P.R. China; 4 Yumei Xu, Department of Chest Disease Center, Zhejiang Rongjun Hospital, 309 Shuangyuan Road, Jiaxing, Zhejiang Province 314000, P.R. China; 5 Gang Lan, Department of Chest Disease Center, Zhejiang Rongjun Hospital, 309 Shuangyuan Road, Jiaxing, Zhejiang Province 314000, P.R. China

**Keywords:** mGPS, Lung cancer, Prognosis, Systemic inflammation, Systematic review, Meta-analysis

## Abstract

**Background & Objective:**

Previous studies have suggested that the modified Glasgow Prognostic Score (mGPS) could be a potential biomarker for lung cancer (LC). However, the association between mGPS and overall survival (OS) or progression-free survival (PFS) in lung cancer patients remains unclear. The purpose of our study was to investigate possible correlation between mGPS and OS or PFS in LC patients.

**Methods:**

An extensive search of PubMed, Cochrane Library, EMbase, Cumulative Index to Nursing and Allied Health Literature (CINAHL), Trip Database, Worldwide Science, and Google Scholar databases was done for relevant articles, published prior to May 30, 2021, that report correlation between mGPS and OS or PFS in LC patients. Pooled hazard ratios (HRs) and 95% confidence intervals (CIs) were used as the main parameters for evaluation.

**Results:**

A total of 28 studies involving 9,748 lung cancer patients were analysed. The pooled analysis revealed that elevated mGPS (≥ 0) was associated with poor OS (HR=1.54; 95% CI, 1.32-1.77) and PFS (HR=1.49; 95% CI, 1.17-1.82). Furthermore, a significant correlation between mGPS (1 or 2) and OS was observed. However, no significant correlation was found between mGPS (1 or 2) and PFS. Subgroup analysis based on ethnicity demonstrated that mGPS ≥ 0 was associated with worse OS compared to mGPS=0 in both Asian (HR=1.46; 95% CI, 1.04-1.89; p<0.05) and Caucasian (HR=1.64; 95% CI, 1.35-1.94; p<0.05) cohorts of LC patients.

**Conclusions:**

Our results demonstrate that positive mGPS is associated with poor survival results. Therefore, mGPS may be used as a biomarker for predicting prognosis in LC patients.

## INTRODUCTION

Lung cancer (LC) is the second most common cancer and a primary cause of cancer-related mortality worldwide.^1^,^2^ LC is often diagnosed at advanced stages, necessitating standard treatments like chemotherapy and surgery.^3^,^4^ Recent advancements in the computed tomography (CT) technology allowed to reduce mortality rates by 16 to 20% in patients with a history of smoking.^5^ Nevertheless, overall survival (OS) for lung cancer patients remains poor. While clinical staging is currently the most reliable prognostic factor for lung cancer, it provides limited information on individual disease progression.

Emerging evidence suggests a correlation between the increased systemic inflammation and the reduced survival rates in various cancer types. Studies have shown that the inflammatory state contributes to angiogenesis, cancer cell proliferation, tumour metastasis, and overall disease progression.^7^,^8^ Serum levels of albumin and C-reactive protein (CRP) serve as established markers for systemic inflammation and nutritional status, respectively.^9^,^10^ The modified Glasgow Prognostic Score (mGPS) that considers both levels of albumin and CRP, currently serves as a prognostic indicator for multiple cancers, including LC.^11^-^14^ Patients with both hypoalbuminemia (<35 mg/L) and increased CRP (>10 mg/L) levels receive a score of Two. A score of One is assigned if either one of these abnormal values is present, while a score of 0 indicates the absence of both abnormalities.

Most studies investigating mGPS have primarily focused on patients who have undergone surgery or chemotherapy.^15^,^16^ Although there is a growing support of the clinical significance of mGPS at different stages of LC,^17^-^24^ the available data is still scarce and inconsistent. Therefore, the predictive capacity of mGPS in lung cancer has not been definitively established. While a study by Jin et al. in 2017 attempted to compile data regarding the predictive efficacy of mGPS for LC, it included only eleven studies, which made the results of the subgroup analyses inconclusive.^25^ Therefore, there is an urgent need for more robust, simple, and easily measurable prognostic indicators to predict LC outcomes.^6^ Hence, the aim of our meta-analysis was to investigate the possible correlation between mGPS and OS or PFS in LC patients.

## METHODS

The meta-analysis was carried out in accordance with the guidelines specified in the Preferred Reporting Items for Systematic Reviews and Meta-Analyses (PRISMA) statement^26^ and was registered with International Prospective Register of Systematic Reviews (PROSPERO) under the identification number CRD42021261007.

Inclusion criteria was published observational studies (prospective or retrospective cohort) investigating the correlation between mGPS and OS or PFS in lung cancer and the studies had patients with confirmed pathologically diagnosed lung cancer. Exclusion criteria were applied to studies that did not report relevant outcomes and those with unavailable full-texts. We performed a thorough search of PubMed, EMbase, Cochrane Library, Trip Database, Worldwide Science, Cumulative Index to Nursing and Allied Health Literature (CINAHL), and Google Scholar databases for relevant articles published up from Jan 1, 1956 to May 30, 2021. The search utilized various combinations of keywords such as “Pulmonary Neoplasms,” “lung cancer,” “Pulmonary Cancer,” “C-Reactive Protein,” “Albumin, Serum,” “Prognosis,” and “modified Glasgow prognosis score”. For each study, two authors conducted independent data extraction that included first author’s name, country of origin, ethnicity, year of publication, cohort type, study duration, patient count, treatment approaches, population characteristics, tumour node metastases (TNM) stage data, endpoints, and survival details.

Newcastle-Ottawa Quality Assessment Scale (NOS)^27^ was used to assess the quality of the included studies. The NOS quality scores range from 0 (lowest) to 8 (highest) based on predetermined criteria. Funnel plot analysis^28^ and Egger’s regression test^29^ were used to assess the publication bias. The survival endpoints for different mGPS scores were analysed using pooled hazard ratios (HRs) with 95% confidence intervals (CIs). Cochran’s Q-test and I^2^-statistic were used to assess heterogeneity. Sensitivity analysis was done by excluding one study at a time from the pooled results to evaluate the robustness of the findings. STATA 12.0 software (Stata Corporation, College Station, TX, USA) was used for statistics.

## RESULTS

**Fig.1:**
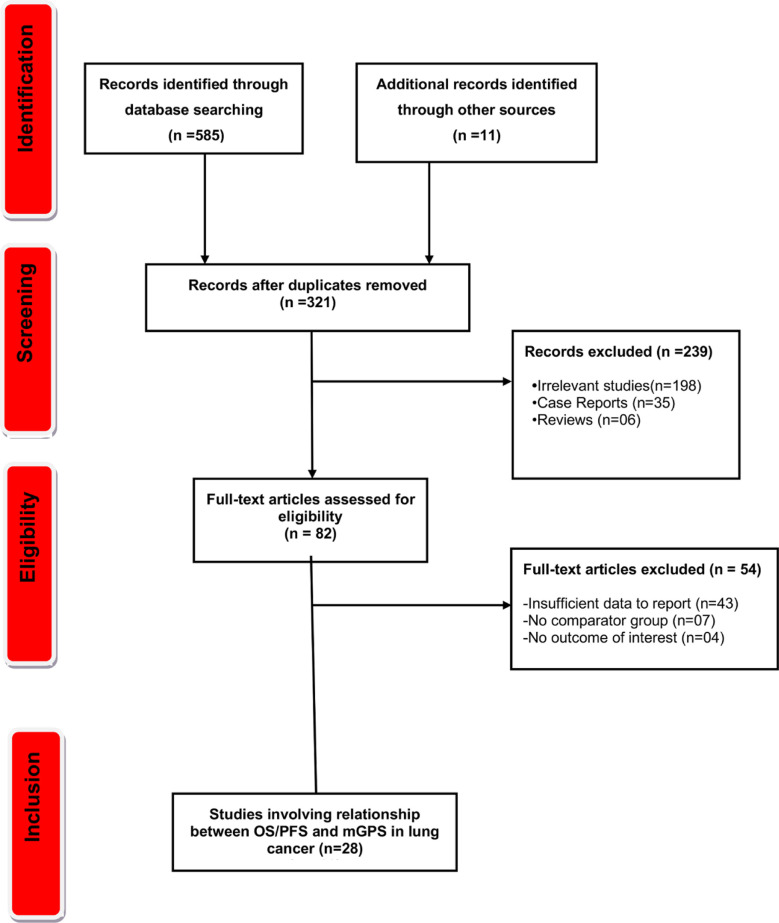
Flow diagram for the selection of studies and specific reasons for exclusion from the present meta-analysis.

A total of 596 records were identified. Of them, 321 remained after duplicates removal. Subsequently, irrelevant studies and review articles were excluded, leaving 82 articles for eligibility assessment. Finally, 28 studies met the eligibility criteria and were included in the meta-analysis. The literature selection process is illustrated in Fig.1.

### Study Characteristics:

The details of the 28 included studies^17^-^24^,^30^-^48^ with 9,748 lung cancer patients are shown in Supplementary Table-I. Of them, 19 were retrospective cohort studies,^21^-^24^,^31^,^32^,^35^,^37^-^46^,^48^ and nine were prospective cohort studies.^17^-^20^,^30^,^33^,^34^,^36^,^47^ Publication years of the studies ranged from 2010 to 2021, and the sample sizes varied from 24 to 1,745 LC patients. One publication was treated as two separate studies due to its coverage of two different cohorts (operative and nonoperative) and separate reporting of hazard ratios (HRs).^22^ Most studies were of good quality, with a Newcastle-Ottawa quality assessment scale (NOS) score of six or higher. Supplementary Table-I.

**Table-I:** Summary of estimates based on subgroup analysis for overall survival (OS) using mGPS in lung cancer patients.

Variables	Subgroups items	No. of studies	Hazard Ratio (95% Confidence Interval)	p* values	Degree of Heterogeneity		References

					I^2^(%)	p* values	
Ethnicity	Asian	9	1.46 (1.04 to 1.89)	<0.05	72.7	<0.0001	71,73,74,74-76,78,89,93
	Caucasian	8	1.64 (1.35 to 1.94)	<0.05	78.6	<0.0001	67-70,72,90-92
Cut-off Value	mGPS=1	8	1.41 (1.28 to 1.60)	<0.05	0	0.64	69,73,75,77,82,84-86
	mGPS=2	11	2.17 (1.71 to 2.64)	<0.05	56.5	0.01	69,73,75,77,80,82,84-88
	mGPS > 0	17	1.54 (1.32 to 1.77)	<0.05	76.8	<0.0001	67-76,78,80,89-92
Therapies	Active/Palliative Care	2	1.74 (1.32 to 2.16)	<0.05	0	0.46	67,68,94-98
	Surgery	4	1.56 (0.81 to 2.52)	0.67	76.6	<0.0001	70,74,75,80
	SBRT	2	1.76 (1.36 to 2.16)	<0.05	0	0.72	71,78
	Chemotherapy	4	1.05 (0.67 to 1.43)	0.54	32.8	0.21	73,74,76,89
	Radiotherapy	2	1.22 (1.11 to 1.33)	<0.05	0	0.51	90,92
	Radiosurgery	1	2.50 (1.32 to 1.77)	<0.05	-	-	91
	Others	1	2.39 (1.85 to 2.93)	<0.05	-	-	69
Pathology	NSCLC	12	1.56 (1.22 to 1.90)	<0.05	73	<0.001	67,68,70,71,74-76,78,80,89,91
	SCLC	2	2.56 (-1.03 to 6.16)	0.16	64.1	0.09	73,90
	NSCLC + SCLC	3	1.70 (1.12 to 2.28)	<0.05	91.7	<0.0001	69,72,92
Study Design	Prospective Cohort	6	1.68 (1.31 to 2.05)	<0.05	82.7	<0.0001	67-70,72,92
	Retrospective Cohort	11	1.47 (1.13 to 1.81)	<0.05	72	<0.0001	71,73,74,74-76,78,80,89-91

**Supplementary Table-I:** Characteristics of included studies in the meta-analysis investigating correlation of mGPS with OS and PFS in lung cancer patients.

S. No.	Author; Year	Country	Ethnicity	Study Period	Cohort Type	Follow up months (median)	Sample Size	Population Type	M/F	mGPS (0/1/2)	TNM Stages (II/III/IV)	Treatment	Endpoints	HR	NOS Score
	Egger *et al.* 2010^67^	UK	Caucasian	Nov 2003-Apr 2004	PC	54	56	NSCLC	34/22	19/31/06	1/29/26	Active/Palliative care	OS	R	8
	Leung *et al.* 2012^68^	UK	Caucasian	May 2001- Nov 2004	PC	83.1	261	NSCLC	107/154	59/163/39	0/134/127	Active/Palliative care	OS	R	9
	Grose *et al.* 2014^69^	UK	Caucasian	2005-2008	PC	24.5	882	NSCLC+ SCLC	487/395	213/290/210	43/188/234	Radical/Palliative	OS	E	9
	Pinato *et al.* 2014^70^	UK	Caucasian	2004-2011	PC	NR	220	NSCLC	110/110	131/39/29	65/29/0	Surgery	OS/RFS	R	9
	Kishi *et al.* 2015^71^	Japan	Asian	Jan 1999- Sep 2010	RC	42	165	NSCLC	120/45	120/45	65/0/0	SBRT	OS	R	7
	Simmons *et al.* 2015^72^	UK	Caucasian	Feb 2006- Oct 2010	PC	12.8	390	NSCLC + SCLC	341/149	103/183/104	NR	Non-operative treatment	OS	E	9
	Zhou *et al.* 2015^73^	China	Asian	Jan 2009- Dec 2011	RC	NR	359	SCLC	304/55	238/110/11	NR	Chemotherapy	OS	R	7
	Fan *et al.* 2016 (Inoperative)^74^	China	Asian	Jan 2011- Dec 2014	RC	20	1745	NSCLC	1217/528	NR	NR	Chemotherapy	OS	R	7
	Fan *et al.* 2016 (Operative)^74^	China	Asian	Jan 2011- Dec 2014	RC	19	1243	NSCLC	713/530	NR	NR	Surgery	OS	R	8
	Osugi *et al.* 2018^75^	Japan	Asian	Jan 2005- Dec 2009	RC	65	327	NSCLC	199/128	301/15/11	232/49/46	Surgery	OS	R	6
	Zhu *et al.* 2016^76^	China	Asian	Upto Nov.2014	RC	28.5	105	NSCLC	72/33	46/47/12	0/20/85	Chemotherapy	OS	R	8
	Freitas *et al.* 2021^76^	Portugal	Caucasian	NR	PC	7	77	NSCLC	55/22	20/11/14	32/26/19	Immunotherapy	OS/PFS	R	9
	Chen *et al.*2021^78^	Japan	Asian	2001-2016	RC	40.7	207	NSCLC	149/58	136/71	58/0/0/0	SBRT	OS/PFS/TP	R	9
	Yamauchi *et al.* 2017^79^	Germany	Caucasian	Jan 2010-Dec 2014	PC	24	156	NSCLC	77/79	66/66/15	29/83/44	Surgery	RFS	R	9
	Matsubara *et al.* 2021^80^	Japan	Asian	Jan 2010-Dec 2015	RC	NR	596	NSCLC	316/280	523/63/10	320/54/55	Surgery	OS/PFS	R	9
	Asakawa *et al.* 2021^81^	Japan	Asian	Apr 2010-July 2017	PC	NR	286	NSCLC	180/106	NR	100/00/00	Surgery	OS/RFS	R	6
	Ni *et al.* 2018 ^82^	China	Asian	Jan 2009-Dec 2015	RC	36	436	NSCLC	297/139	NR	NR	Chemotherapy	OS	R	7
	Minami *et al.* 2017^83^	Japan	Caucasian	Nov 2007-June 2016	RC	NR	97	SCLC	77/20	66/20/11	NR	Chemotherapy	OS/PFS	R	6
	Sonehara *et al.* 2R019^84^	Japan	Asian	Jan 2015-Dec 2018	RC	13.6	83	SCLC	70/13	43/24/16	0/6/77	Chemotherapy	OS	R	8
	Zhou *et al.* 2019^85^	China	Asian	Jan 2006-Dec 2011	RC	15.6	451	SCLC	389/62	273/162/16	158/36/0	Chemotherapy	OS	R	8
	Kurishima *et al.* 2017^86^	Japan	Asian	Apr 1999- July 2006	RC	NR	319	SCLC	273/46	192/54/73	NR	Chemotherapy	OS	R	6
	Takamori *et al*. 2021^87^	Japan	Asian	Jan 2016- Dec 2019	RC	13.7	304	NSCLC	242/62	137/57/110	0/51/202	ICIs Monotherapy	OS/PFS	R	7
	Araki *et al.* 2020^88^	Japan	Asian	Jan 2015-Dec 2019	RC	NR	113	NSCLC	87/26	39/37/37	NR	Chemotherapy	OS/PFS	R	6
	Ogura *et al.* 2021^89^	Japan	Asian	Feb 2019-July 2020	RC	NR	34	NSCLC	29/5	10/21	NR	Chemotherapy	OS/PFS	R	5
	Dolan *et al.*2020^90^	UK	Caucasian	June 2008-Dec 2012	RC	NR	119	SCLC	67/62	58/20/41	22/55/0	Radical Radiotherapy	OS	R	8
	Cho *et al.* 2021^91^	Austria	Caucasian	2012-2018	RC	NR	49	NSCLC	26/23	23/14/12	NR	Radiosurgery	OS	R	6
	Abbass *et al.* 2020^92^	UK	Caucasian	Jan 2009- Feb 2017	PC	10	644	NSCLC + SCLC	330/313	169/175/299	0/240/403	Radiotherapy	OS	R	9
	Matsubara *et al.* 2020^93^	Japan	Asian	Jan 2018-Mar 2019	RC	NR	24	NSCLC	17/17	13/11	NR	Chemotherapy	OS	R	5

***Abbreviations:*** PC- Prospective Cohort; R -Retrospective Cohort; OS- Overall Survival; RFS- Recurrence-free survival; PFS- Progression free survival; NR- Not Reported; SBRT-Stereotactic body radiation therapy; TP-Time to Progression; ICIs-immune checkpoint inhibitors; mGPS-modified Glasgow prognostic score; NSCLC-Non-small cell lung Cancer; SCLC- Small cell lung Cancer; R-Reported in included article; E-Estimated from Kaplan Meir Curves.

### Association between mGPS and OS:

**Fig.2:**
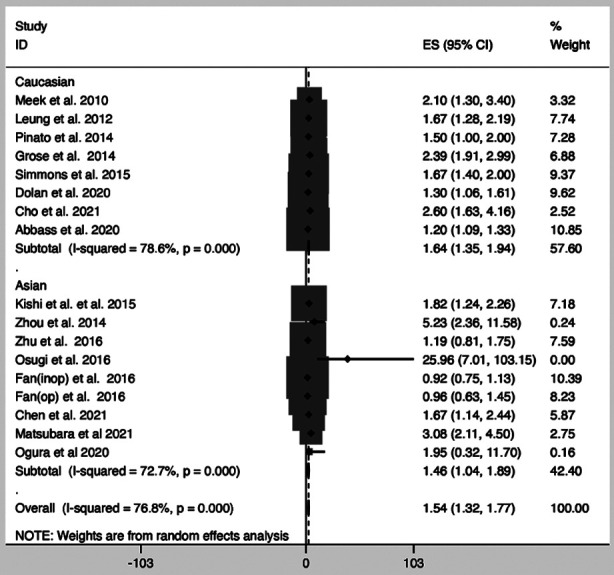
Forest plot of the association between modified Glasgow prognostic score (mGPS) and overall survival (OS) in patients with lung cancer.

A meta-analysis incorporating 17 studies^17^-^24^,^30^-^32^,^35^,^44^-^47^ demonstrated a significant association between elevated mGPS and poor OS in LC patients (HR=1.54; 95% CI, 1.32-1.77). (Fig.2) Additionally, there was a significant association of mGPS of 1 and OS (HR=1.41; 95% CI, 1.28-1.60), as well as between an mGPS of 2 and OS (HR=2.17; 95% CI, 1.71-2.64). Table-I

### Subgroup Analyses for the Association between mGPS and OS:

Subgroup analyses were conducted to explore the relationship between OS and mGPS based on ethnicity, pathology, study design, therapies, and different mGPS scores. Table-I Consistently, studies with both prospective and retrospective cohorts indicated a significant correlation between mGPS > 0 and OS. In terms of ethnicity, subgroup analysis showed that mGPS ≥ 0 was associated with worse OS compared to mGPS=0 in both Asian (HR=1.46; 95% CI, 1.04-1.89) and Caucasian (HR=1.64; 95% CI, 1.35-1.94) populations. Stratifying patients by treatment revealed a similar trend for those undergoing active care (HR=1.74; 95% CI, 1.32-2.16), stereotactic body radiation therapy (SBRT) (HR=1.76; 95% CI, 1.36-2.16), and radiosurgery (HR=1.56; 95% CI, 1.22-1.90).

Stratifying patients based on pathology (Table-I) showed a significant correlation between mGPS ≥ 0 and OS in cases of non-small cell lung cancer (NSCLC) (HR=1.56; 95% CI, 1.22-1.90). Furthermore, both NSCLC and small cell lung cancer (SCLC) patients presented a significant correlation between mGPS ≥ 0 and OS (HR=1.70; 95% CI, 1.12-2.28). However, no correlation was found between mGPS ≥ 0 and OS in patients with SCLC (HR=2.56; 95% CI, -1.03-6.16).

### Association between mGPS and PFS:

**Fig.3:**
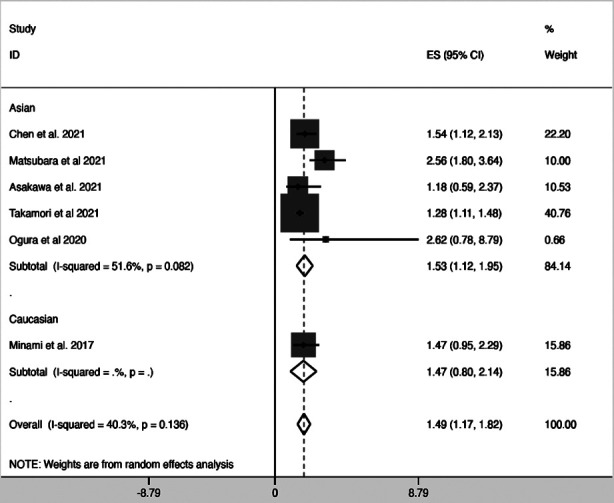
Forest plot of the association between modified Glasgow prognostic score (mGPS) and progression free survival (PFS) in patients with lung cancer.

Only six studies^24^,^35^,^36^,^38^,^42^,^44^ provided HRs and 95% CIs for the link between mGPS ≥ 0 and PFS in lung cancer patients. The pooled data revealed a significant association between mGPS > 0 and PFS (HR=1.49; 95% CI, 1.17-1.82). Fig.3 However, no significant correlation was observed between an mGPS score of 1 and PFS (HR=1.33; 95% CI, 0.72-1.94) or an mGPS score of Two and PFS (HR=1.92; 95% CI, 0.57-3.27). Table-II.

**Table-II:** Summary of estimates based on subgroup analysis for progression free survival (PFS) using mGPS in lung cancer patients.

Variables	Subgroups items	No. of studies	Hazard Ratio (95%Confidence Interval)	p* values	Degree of Heterogeneity		References

					I^2^(%)	p* values	
Ethnicity	Asian	5	1.53 (1.12 to 1.95)	<0.05	51.6	0.08	71,73-76,78,81,87,89,93
	Caucasian	1	1.47 (0.80 to 2.14)	0.87	-	-	83
Cut-off Value	mGPS=1	2	1.33 (0.72 to 1.94)	0.43	0	0.90	77,79
	mGPS=2	2	1.92 (0.57 to 3.27)	0.34	0	0.31	77,79
	mGPS > 0	6	1.49 (1.17 to 1.82)	<0.05	40.3	0.13	78,80,81,83,87,89
Therapies	Surgery	2	1.86 (0.51 to 3.22)	0.26	77.6	0.03	80,81
	SBRT	1	1.54 (1.03 to 2.05)	< 0.05	-	-	78
	Chemotherapy	2	1.50 (0.84 to 2.16)	0.31	0	0.57	83,89
	ICIs Monotherapy	1	1.28 (1.10 to 1.47)	<0.05	-	-	87
Pathology	NSCLC	6	1.49 (1.17 to 1.82)	<0.05	40.3	0.13	78,80,81,83,87,89
	SCLC	0	-	-	-	-	-
	NSCLC + SCLC	0	-	-	-	-	-
Study Design	Prospective Cohort	-	-	-	-	-	-
	Retrospective Cohort	6	1.49 (1.17 to 1.82)	<0.05	40.3	0.13	78,80,81,83,87,89

### Subgroup analyses for the association between mGPS and PFS:

Subgroup analysis based on ethnicity revealed that in the Asian population, patients with mGPS ≥ 0 had poorer PFS compared to patients with mGPS=0 (HR=1.53; 95% CI, 1.12-1.95). No such correlation between mGPS and PFS was detected in the Caucasian population (HR=1.47; 95% CI, 0.80-2.14). Stratified analysis based on pathology. Table-II demonstrates a significant correlation between mGPS and PFS in patients with NSCLC (HR=1.49; 95% CI, 1.17-1.82).

### Sensitivity analysis and publication bias:

A sensitivity analysis was conducted by sequentially excluding one study at a time from the pooled results. The analysis demonstrated that the combined HRs for both OS and PFS did not exhibit significant variations, indicating the stability of the results. Egger’s test indicated evidence of publication bias for the correlation between mGPS > 0 and OS (P<0.001). However, no publication bias was observed for the correlation between mGPS > 0 and PFS (P=0.57).

## DISCUSSION

The results of our meta-analysis, which included 28 studies, indicate that an elevated mGPS is a predictive indicator of poor survival in LC patients. Subgroup analyses further supported the predictive effectiveness of mGPS for OS and PFS in lung cancer. Our findings suggest that mGPS may serve as a reliable and cost-effective prognostic marker for LC. While previous meta-analysis have primarily focused on assessing the prognostic value of mGPS in relation to OS.^25^ Our study represents the first meta-analysis to provide comprehensive pooled evidence regarding the prognostic value of mGPS for both OS and PFS in LC patients.

Inflammatory state plays a critical role in maintaining tumor microenvironment as it enables cancer cells to evade the immune system.^49^ Studies showed that inflammatory markers, such as the neutrophil/lymphocyte and platelet/lymphocyte ratios, have shown correlations with poor prognosis in certain solid malignancies.^7^,^15^ The modified Glasgow Prognostic Score takes into account both serum C-reactive protein (CRP) and albumin levels in clinical samples,^33^,^50^ and reflects both the inflammatory and nutritional status of patients.^51^ Production of CRP, an acute-phase protein primarily produced by hepatocytes, is triggered by inflammation, tissue injury, and infection. Elevated CRP levels beyond a specific threshold have been associated with unfavorable survival outcomes in various malignancies, including lung cancer.^52^-^56^

Similarly, hypoalbuminemia is linked to tumor progression and poor survival in lung cancer.^9^,^57^ Furthermore, it has been observed that as CRP levels increase, albumin levels tend to decline in cancer patients, suggesting a connection between systemic inflammation and cachexia.^58^ It was shown in a recent study by Ran et al. (2022) that a number of factors affect the prognosis and survival of elderly patients with advanced NSCLC.^59^ Age, performance status score, smoking history, and style of treatment are some of these variables. It is essential to give efficient therapies in accordance with the tenets of evidence-based medicine in order to guarantee the best results. Similar to the previous study, Li et al.^60^ further investigation on the treatment results of patients with advanced NSCLC and EGFR gene mutations. When compared to Gefitinib medication in this patient population, the study found that oxitinib treatment was linked with considerably longer PFS and a reduced risk of adverse events. These results highlight the significance of identifying the best therapeutic strategy for each patient based on their unique genetic traits and therapeutic response.

Several previous meta-analyses have examined the prognostic value of mGPS in different types of cancer.^61^-^65^ For example, Nie et al. conducted an 11-study meta-analysis involving 2,830 patients and demonstrated that mGPS is a predictor of poor OS and PFS in gynecologic malignancies.^65^ Another study found that a positive mGPS is associated with lower OS and cancer-specific survival (CSS) in colorectal cancer.^66^ Additionally, a recent meta-analysis of 20 studies indicated that mGPS may independently predict outcomes in patients with urological cancer.^63^ Our results are consistent with these previous findings, providing further evidence that elevated mGPS can be used as a prognostic marker for OS in lung cancer. Future research efforts should focus on the development of specific prognostic models or nomograms that incorporate mGPS as a pivotal prognostic factor for LC. This personalized approach can significantly enhance prognosis assessment and guide treatment decisions more effectively. By integrating mGPS assessment and considering the specific needs of patients, healthcare providers can make more informed decisions, ultimately enhancing the patient’s journey through diagnosis, treatment, and recovery.

### Limitations of the study:

Firstly, 19 out of 28 included studies were retrospective cohort studies, which may introduce inherent biases. Secondly, the participants in the included studies were at different stages of lung cancer progression, which could introduce variability in the results. Thirdly, due to limited data availability in the included studies, we were unable to develop a prognostic model specifically for patients with lung cancer.

### Recommendation:

Further studies should focus on the development of a specific predictive model or nomogram that incorporates the mGPS as a prognostic factor for lung cancer. This would provide a more comprehensive and personalized approach to prognosis assessment in LC patients.

## CONCLUSION

We have shown that mGPS may serve as a useful biomarker for predicting prognosis in lung cancer patients, with a positive mGPS linked to poor survival.
